# Perspective taking in the human brain: complementary evidence from neuroimaging studies with media-based naturalistic stimuli and artificial controlled paradigms

**DOI:** 10.3389/fnhum.2023.1051934

**Published:** 2023-02-15

**Authors:** Iiro P. Jääskeläinen, Vladimir Kosonogov

**Affiliations:** ^1^Brain and Mind Laboratory, Department of Neuroscience and Biomedical Engineering, Aalto University School of Science, Espoo, Finland; ^2^International Laboratory of Social Neurobiology, Institute of Cognitive Neuroscience, HSE University, Moscow, Russia

**Keywords:** perspective taking, human brain, neuroimaging, precuneus, temporoparietal junction, prefrontal cortex

## Abstract

Perception and interpretation of various types of events and information in life crucially depend on one’s perspective. A specific perspective can be explicitly adopted, for example, *via* instructing an experimental subject, implicitly *via* *a priori* information given to subjects, and by subjects’ personality traits or cultural background. The neural basis of perspective taking has been addressed in a number of recent neuroimaging studies, some of which have used movies and narratives as media-based stimuli to pursue a holistic understanding of the phenomenon under ecologically valid conditions. Results across these studies suggest that the human brain flexibly adapts to support the information-processing needs of different perspectives, however, also that inferior temporal-occipital areas and posterior-medial parietal areas are engaged across different perspectives. These findings are complemented by studies that have investigated specific aspects of perspective taking with highly controlled experimental designs. They have disclosed involvement of the temporoparietal junction in visual perspective taking and the importance of the affective component of the pain matrix when empathizing with others’ pain. Identification with the protagonists also seems to matter, as dorsomedial vs. ventromedial prefrontal areas are recruited when the protagonist is dissimilar vs. similar to self. Finally, as a translational aspect, perspective taking can, under certain conditions, serve as an effective emotion regulation technique, wherein lateral and medial regions of the prefrontal cortex seem to support reappraisal processes. Together, findings from studies with media-based stimuli and more traditional paradigms complement each other to gain a comprehensive understanding of the neural basis of perspective taking.

## Introduction

As famously phrased by Obi-Wan Kenobi, the fictional Jedi-master character in Star Wars “*many of the truths we cling to depend greatly on our own point of view*” (Return of the Jedi, Lucasfilm Ltd., 1983). Indeed, the same event can be perceived differently by viewers, depending on, for example, their goals, *a priori* information, their personalities, and their cultural backgrounds. In general, perspective taking is an important and multifaceted human social-cognitive ability. It can refer to emotionally putting oneself into the shoes of others (e.g., “feeling the other’s sadness”), cognitively assessing how another person could see a particular situation (e.g., “based on what he suspects, it is no wonder that he is worried”), adopting of an expert point of view (e.g., an architect seeing an urban environment differently from a layperson), as well as cognitively reappraising one’s situation (e.g., re-thinking a feared outcome as not realistic). Perspective can be taken either explicitly, for example, when a subject adopts a particular perspective, or implicitly, for example, when *a priori* information or one’s cultural background guides one to look at an event from a particular point of view. Perspective taking has translational utility, as it is widely utilized in psychotherapy (Clark, 2023) and in interventions to alleviate inter group tensions (Halperin et al., 2013).

**Figure 1:**
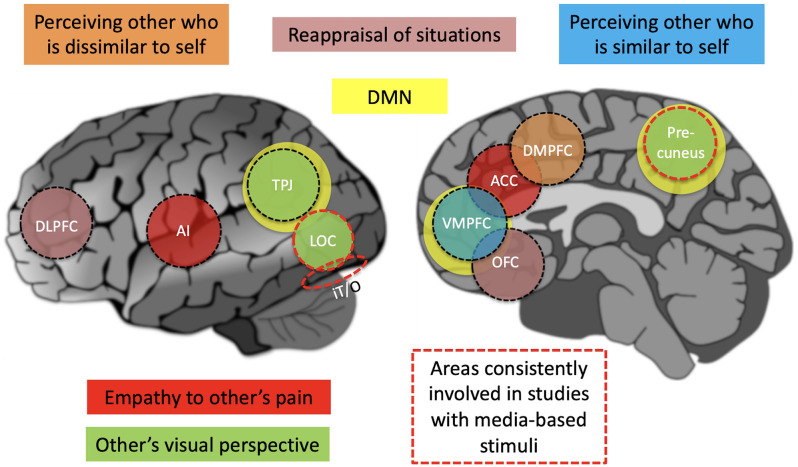
A simplified summary schematic of brain regions that are involved in perspective-taking tasks. Areas consistently involved in studies with media-based stimuli include the precuneous, LOC, and IT/O, with the precuneus also being an important part of the DMN that is plotted with yellow color. Areas involved with specific perspectives, such as empathy to other’s pain, are plotted with different colors. ACC, anterior cingulate cortex; AI, anterior insula; DLPFC, dorsolateral prefrontal cortex; DMN, default mode network; DMPFC, dorsomedial prefrontal cortex; IT/O, inferior temporal/occipital cortex; LOC, lateral occipital cortex; OFC, orbitofrontal cortex; TPJ, temporoparietal junction.

Elucidation of the neural basis of perspective taking has been the quest in an increasing number of neuroimaging studies. Some have utilized dynamic media-based stimuli such as movies that subjects have viewed from different perspectives. The strength of this approach, facilitated by development of new data analysis approaches, is that perceptual, cognitive, and emotional functions can be engaged in subjects in more ecologically valid ways (Hasson et al., 2010; Jääskeläinen et al., 2021). In general, a pattern of results suggests that there are both brain areas specific to the particulars of a given perspective taking task and brain areas that participate across different perspective taking tasks. In addition, there are a number of studies wherein specific instances of perspective taking have been scrutinized using highly controlled experimental designs. These studies have pinpointed neural mechanisms involved in, for example, feeling the emotions and when adopting the visual perspective of another person. We will go over the findings of these studies, starting with the more holistic results obtained in the studies with media-based naturalistic stimuli. This will be followed by going over the more specific findings from studies that have utilized more controlled tasks, as these offer cues into why certain areas might be engaged when we take perspective under more naturalistic conditions. These findings are summarized in Figure 1 and Table 1.

**Table 1:** Summary of studies with different types of perspective taking studies.

Study	Task, stimuli	Brain measure	Brains areas involved
Allard and Kensinger (2014)	Selective attention and reappraisal emotion regulation during emotional movie clips	BOLD activity in differences by conditions and age groups (young vs. old)	PCC, IFG, OFC, VLPFC, ACC, DLPFC, DMPFC
Bacha-Trams et al. (2017)	Watching a movie thinking sister-protagonists in a moral dilemma are genetics vs. adopted	BOLD ISC differences genetic vs. adopted viewing conditions	ACC, VMPFC, IFG, DLPFC, IN, PCC, Precun, Cun, FG, MOG, STG, STS,
Bacha-Trams et al. (2020)	Watching a movie from perspective of organ donor vs. recipient protagonists	BOLD ISC differences between to-be-organ donor vs. -recipient perspectives	DLPFC, IFG, PMC, Precun, PCC, Cal, FG, Cun, IOG, MOG, AG, TPJ, TP, STG
Bierzynska et al. (2019)	Subjects with different temperamental traits watching arousing sports movie clips	BOLD actitivity correlating with temperamental traits	MPFC, MFG, IFG, SFG, Precun, HC, IN, LG, AMG, CN
Bruneau and Saxe (2010)	Subjects of two ethnic gropus in conflict presented with statements supporting one vs. other	BOLD activity difference between groups explained by intergroup views	Precun, PCC, TPJ
Cao et al. (2020)	Watching pictures with emotional reappraisal: subjects divided based on reappraisal success	Event-related brain potential measures to pictures during successful reappraisal	N/A, frontal-central late positive potential
Dörfel et al. (2014)	Four emotion regulation strategies during watching of negative-emotional pictures	BOLD activity difference in regulation vs. neutral viewing conditions	AG, MFG, SFG, SMA, Precun, MCC, MTG, STG, cer, OFC, IN, IFG, TP, caudate, putamen, PCG
Dumontheil et al. (2010)	Visual perspective with consideration of other’s intentionality	BOLD activity during visual perspective with intentionality inference	MPFC, IFG, MTG, TP
Hakonen et al. (2022)	Subjects with two dfferent family cultural backgrounds listening to an audiobook	BOLD ISC differences between the two cultural-background groups	PCun, PCC, SPL, MOG, LOC, MTG, Cun, LG
Jackson et al. (2005)	Subjects watching others in pain vs. neutral pictures	BOLD activity difference when seeing others in pain vs. neutral pictures	ACC, IN, PPC, thalamus, cerebellum
Jacob et al. (2018)	Movie clips eliciting anger in experimental subjects who differed in emotional regulation traits	BOLD based depdency network analysis to disclose the central network node in anger regulation	VMPFC
Kaiser et al. (2008)	Subjects taking third-person perspective of an avatar arranging items	BOLD activity difference when taking third vs. first person perspective	IFG, SFG, SMA, PCG, IN, Precun, MOG, Cerebellum
Koenigsberg et al. (2010)	Viewing pictures of neutral vs. aversive social scenes passively vs. during emotional distancing	BOLD activity differences between conditions	Amygdala, Precun, PCC, IPS, MTG, STG, dACC, MPFC, LPFC
Lahnakoski et al. (2014)	Detective vs. decorator perspectives during video viewing	ISC of BOLD differences between perspectives as well as within vs. across perspectives ISC differences	PPC, LOC, PHG
Langeslag and Surti (2017)	Emotional reappraisal of negative-valenced emotional pictures	Event-related brain potentials to negative pictures during emotional reappraisal	N/A, frontal-central late positive potential
Mano et al. (2009)	Subjects presented with sentences with protagonist “here” vs. “there”	BOLD activity difference for spatially coupled (“here”) vs. decoupled (“there”)	Precun, TPJ
Mitchell et al. (2006)	Photographs of persons similar vs. dissimilar to self in political views	BOLD activity difference between photos of persons similar vs. dissimilar to self	VMPFC, DMPFC
Powers et al. (2020)	Viewing aversive pictures during different types of emotional distancing	BOLD activity differences between conditions	SPL, MTG, STS, STG, IFG, SFG, Precun, AG, ITG, MFG, FG, SMG, IPL, PCC
Singer et al. (2004)	Subjects watching loved one in pain vs. feeling pain themselves	BOLD activity difference when seeing loved one in pain vs. experiencing pain	ACC, IN, cerebellum, brain stem
Sonkusare et al. (2020)	Emotional pictures, music, and movies	Intracranial EEG recorded in temporal and amygdala to explore functional connectivity	TP, amygdala
Sulpizio et al. (2021)	Viewing negative vs. neutral pictures and words during emotional distancing	EEG oscillatory theta and beta measures	N/A, posterior EEG theta activity
van der Heiden et al. (2013)	Subjects adopting perspective of other vs. self in pain when looking at limbs in pain in pictures	BOLD activity difference when adopting other vs. self in pain pespective	IFG, SMA, MFG, IFO, MTG, LG
Vogeley et al. (2004)	Subjects taking third-person perspective of an avatar arranging items	BOLD activity difference when taking third vs. first person perspective	MPFC, IFG, Precun, IPL, IOG, Cerebellum
Yeshurun et al. (2017)	Listening to an audiobook thinking the protagonist rigthly suspects cheating vs. is jealous	Euclidian distance in BOLD time series with difference in interpretation	DMPFC, VMPFC, PMC, VLPFC, Precun, PCC, TPJ, Thal

*Abbreviations: ACC, anterior cingulate cortex; AG, angular gyrus; cer, cerebellum; Cun, cuneus; FG, fusiform gyrus; dACC, dorsal anterior cingulate cortex; DLPFC, dorsolateral prefrontal cortex; DMPFC, dorsomedial prefrontal cortex; IFG, inferior frontal gyrus; IN, insula; IFO, inferior frontal operculum; IOG, inferior occipital gyrus; ITG, inferior temporal gyrus; LG, lingual gyrus; LPFC, lateral prefrontal cortex; LOC, lateral occipital cortex; MCC, middle cingulate gyrus; MFG, middle frontal gyrus; MPFC, medial prefrontal cortex; MOG, middle occipital gyrus; MTG, middle temporal gyrus; OFC, orbitofrontal gyrus; PCC, posterior cingulate gyrus; PHG, parahippocampal gyrus; Precun, precuneous; SFG, superior frontal gyrus; SMA, supplementary motor area; SPL, superior parietal lobule; STG, superior temporal gyrus; STS, superior temporal sulcus; Thal, thalamus; TP, temporal pole; TPJ, temporoparietal junction; VLPFC, ventrolateral prefrontal cortex; VMPFC, ventromedial prefrontal cortex*.

## Perspective taking modulates processing of media-based naturalistic stimuli

There are an increasing number of studies that have presented experimental subjects with media-based stimuli (i.e., movies and narratives) during neuroimaging while the subjects have been either explicitly asked or implicitly guided to adopt different perspectives. Such studies have offered important clues into how the brain filters information under ecologically valid, complex and dynamic conditions based on the type of perspective.

In an early study, subjects watched a 10-min clip from the perspectives of a forensic detective (to figure out who is guilty of murder) vs. interior/exterior decorator (to assess how to improve the interiors and exteriors shown in the clip; Lahnakoski et al., 2014). Between-condition differences were assessed using inter-subject correlation (ISC) of brain activity. The ISC compares the response time course in each brain region from one subject to the response time courses obtained in the same brain region from other subjects, identifying brain regions with similar responses, and, thus, also of shared processing and/or understanding across subjects (Hasson et al., 2008; Yeshurun et al., 2021). It was calculated for each voxel as the average Pearson correlation coefficient of brain activity time series across all pairs of subjects (Hasson et al., 2004; Kauppi et al., 2010). The detective perspective, in general, induced higher ISC of brain activity, including in precuneus, than the decorator perspective, which may reflect that the subjects were more idiosyncratic in how they adopted the decorator perspective. Within-perspectives vs. between-perspectives ISC analysis showed higher within-perspectives ISC in lateral occipital areas and in posterior parietal areas that are part of the dorsal attention network (DAN; Corbetta and Shulman, 2002), thought to be active when a person is engaging attention in the external environment, as well as in posterior hippocampus (Lahnakoski et al., 2014). Naturally, interpreting this result, one has to keep in mind that the task involved contrasting social (i.e., subjects paying attention to the protagonists for suspicious behaviors) vs. non-social (i.e., subjects paying attention to decorations) perspectives.

Subsequent studies have contrasted two social perspectives. In one such study, individuals listened to an audiobook, thinking that a protagonist exhibited unfounded vs. justified jealousy. Between-condition differences were computed as Eucli dean distances (i.e., as in case of ISC, how similar the BOLD signal was in different conditions). They were observed in the temporo-parietal junction (TPJ), superior temporal sulcus (STS), hippocampus, thalamus, precuneus, premotor cortex (PMC), dorsomedial prefrontal cortex (DMPFC), ventrolateral prefrontal cortex (VLPFC), and ventromedial prefrontal cortex (VMPFC; Yeshurun et al., 2017). Notably, many of these areas overlap with specific functional brain networks. The TPJ, precuneus and VMPFC overlap the default-mode network (DMN) that is activated when the brain is at rest, however, also during watching of social interactions . This suggests that the DMN activation at rest might be due to mind wandering about social interactions (Iacoboni et al., 2004). The VLPFC and PMC overlap the mirror neuron system (MNS), which is activated both when producing and observing actions by others (Rizzolatti, 2005). The VLPFC as well as STS, in turn, overlap a network of areas associated with language processing (Friederici and Gierhan, 2013). Overall, the results can be understood from the perspective that the two conditions differently drove mentalization that would recruit brain areas supporting theory-of-mind, as well as shape the understanding of the plot for which DMN structures seem to be important, in particular the precuneus (Nguyen et al., 2019).

In another study, subjects watched a movie, thinking that the main protagonists were either genetic or adopted sisters. Higher ISC was observed in the “genetic” condition in several areas overlapping the DAN (superior parietal areas) and the DMN (VMPFC, precuneus, and posterior cingulate cortex), as well as other regions including inferior and lateral prefrontal, superior temporal, and occipital areas (Bacha-Trams et al., 2017). Especially the medial DMN structures suggest that the enhanced ISC was due to more similar mentalization and plot-interpretation, whereas ISC in the DAN suggest a more similar deployment of attention, though it is important to keep in mind the caveats of reverse inference (Poldrack, 2011). Finally, a recent study contrasted adopting two social perspectives, as subjects were instructed to put themselves into the shoes of a potential organ donor vs. recipient sister in a movie depicting a moral dilemma of a healthy sister refusing to donate her kidney to save her sick sister. Even though, the differences in ISC were distributed across the brain, the results showed the engagement of empathy circuitry, including the anterior insula (AI), anterior cingulate cortex (ACC) and somatosensory areas, in the perspective of the sick sister (Bacha-Trams et al., 2020). As for the perspective of the healthy sister, they highlighted the role of moral dilemma processing areas, specifically DLPFC and inferior frontal gyrus (IFG; Bacha-Trams et al., 2017).

In addition to explicit perspective-taking tasks and implicit *a priori* information, such factors as cultural family background and personality traits can shape the perspective from which one interprets and feels the world. In a recent study, subjects with two different family cultural-ethnic backgrounds listened to a 71-min audiobook during fMRI. Differences in ISC between the two groups were noted in lateral aspects of the temporal lobe, LOC, visual areas, posterior cingulate cortex, and precuneus. This suggests that the cultural background, in subtle ways, influenced how the audiobook was heard at the level of single word semantics, visual imagery elicited by the story, and at the level of interpretation of the plot of the audiobook (Hakonen et al., 2022). Behavioral findings paralleled these neuroimaging findings as the subject groups exhibited significant differences in word-lists that they produced in order to describe what had been on their minds while they heard the audiobook during neuroimaging (Hakonen et al., 2022). As other findings of note, it was observed that the precuneus exhibited higher activity during exposure to statements exhibiting pro-outgroup than pro-ingroup viewpoints in members of groups involved in an intergroup conflict (Bruneau and Saxe, 2010). Finally, temperamental traits have been noted to modulate brain activity when watching arousing sports movie s (Bierzynska et al., 2019).

Overall, the studies with ecologically valid media-based stimuli suggest that the human brain flexibly adapts to the specific information-processing needs of the perspectives. However, inferior temporal-occipital areas and posterior-medial parietal areas seem to be quite consistently recruited across different perspectives. One drawback of using media-based stimuli is that it is difficult to discern which specific factors resulted in recruitment of specific brain areas. Fortunately, there are studies that have studied this question by looking at specific factors in isolation. These will be reviewed next.

## Neural correlates of visual perspective taking

Visual perspective taking, the ability to see the visuospatial world from the perspective of another person, can be seen as a basic mechanism that supports higher-level perspective taking. Visual perspective taking has been studied, for example, with tasks where an avatar is surrounded by objects and the subjects are to assess the spatial arrangement of the objects (e.g., which one is ahead of the other) from third-person vs. first-person perspectives, and/or manipulate objects taking into account what the avatar is seeing. In a study where subjects adopted a visual perspective of an avatar relative to surrounding objects, increased activity was observed in precuneus, bilateral frontal areas, cerebellum, as well as left-hemisphere temporal and occipitoparietal areas (Vogeley et al., 2004). These findings pinpointed areas specifically involved in adopting the third-person visual perspective. The involvement of these areas was confirmed in a subsequent study (Kaiser et al., 2008). In addition, spatial perspective-taking (a protagonist in a narrative being “here” vs. “there”) was observed to be linked to temporoparietal junction (TPJ) and posterior medial parietal areas (Mano et al., 2009). Finally, a transcranial-current stimulation study causally confirmed the involvement of TPJ bilaterally in visual perspective taking (Santiesteban et al., 2015). Taken together, these findings indicate brain regions that are recruited when taking the visual-spatial perspective of another person (see **Figure 1**). These results were extended in a study wherein visual perspective was adopted to take into account the intentions of another, resulting in activation of the dorsal medial prefrontal cortex (DMPFC) as well as IFG and temporal pole (TP), indicated as important for social cognition (Dumontheil et al., 2010).

## Empathetic perspective-taking

Empathy refers to feeling the emotions of others, including their pain. This is one of the most researched areas of perspective taking. In pioneering studies, it was shown that seeing one’s loved one in pain activated the AI and ACC that are associated with affective components of pain, yet failed to activate somatosensory and caudal cingulate cortex that were activated when feeling pain oneself (Singer et al., 2004; Jackson et al., 2005). These findings suggest that these areas are involved when adopting an empathetic perspective towards others’ suffering.

In another study, adopting empathetic self-perspective vs. other-perspective, to pictures of hands and feet in pain, resulted in elevated activity in the left supramarginal gyrus. In the reverse contrast, other-perspective vs. self-perspective, stronger activity was seen in the dorsolateral prefrontal cortex (DLPFC), ventrolateral prefrontal cortex (VLPFC), middle cingulate, pallidum, as well as the superior temporal sulcus (STS; van der Heiden et al., 2013). The more wide-spread activation and delayed hemodynamic responses, when looking at the pictures empathizing with others’ pain, were taken to suggest that adopting the other-perspective is more effort-demanding than the self-perspective (van der Heiden et al., 2013).

Researchers often connect empathetic perspective taking with the MNS, which is activated both during one’s own actions and the perception of others’ actions (Rizzolatti, 2005). A recent meta-analysis confirms that emotional and cognitive empathy are moderately correlated with MNS activity (Bekkali et al., 2021). Together, these findings suggest that taking an empathetic perspective to others’ suffering, we observe a pattern of brain activity that partially overlaps with what is seen when we feel pain ourselves (see Figure 1).

## Perspective taking when the other is viewed as similar vs. dissimilar to self

There are neuroimaging findings indicating that different brain mechanisms are utilized when mentalizing about others who are similar to self vs. others who are dissimilar to self (Mitchell et al., 2006). Seeing others as similar to self can result from identifying shared group membership (e.g., national, ethnic, religious, political, place of work, or supporters of the same sports team). Lack of such identification, or seeing the other as belonging to another group, would result in one seeing the other as dissimilar to self. It seems, then, that taking the perspective of similar vs. dissimilar others relies on differential brain mechanisms. Interestingly, DMPFC areas implicated in cases of the perception of dissimilar others partially overlap the DMPFC activation elicited when adopting the visual perspective of an avatar (Kaiser et al., 2008). In contrast, the VMPFC activity during the mentalization about others similar to self coincided with areas engaged during autobiographical memory recall. Thus, in the case of watching movies, the perceived similarity of the protagonists to oneself likely modulates the neural mechanisms that are recruited in a perspective taking task.

## Emotion regulation *via* perspective taking

Emotion regulation refers to attempts to influence emotions in ourselves (or others) and encompasses up- and down-regulation of positive and negative emotions (McRae and Gross, 2020). Coping is a similar term, limited to down-regulation of negative emotions (Lazarus and Folkman, 1984). Cognitive reappraisal is one of the most effective emotion regulation strategies (Kalokerinos et al., 2017; Mohammed et al., 2021). Using this strategy, individuals consider other perspectives and potential antecedents to change how they perceive or interpret events and, consequently, regulate their emotions, be it up- or down-regulation (Wang et al., 2017). For example, identifying with transgressors through perspective taking facilitates forgiveness (Menahem and Love, 2013). Specific manuals have been elaborated to control anger using perspective-taking (Day et al., 2008). Narrative approaches are especially effective for perspective-taking skills (Bamberg, 1991).

In laboratory studies, instructions for emotion regulation are typically given before a stimulus which would provoke a strong negative (or positive) emotion. EEG studies showed an increased late positive potential amplitude in the reappraisal condition (Langeslag and Surti, 2017; Cao et al., 2020) and stronger frontal EEG activity associated with better emotion regulation (Dennis and Solomon, 2010). Cao et al. (2021) demonstrated that TMS over the left VLPFC shifted valence ratings in a more positive direction during positive reappraisal. Dörfel et al. (2014), using fMRI, showed that reappraisal of negative stimuli involved the left VLPFC and orbitofrontal gyrus. Allard and Kensinger (2014) observed activation of lateral (DLPFC, VLPFC, OFC) and medial prefrontal cortex (ACC), while subjects hedonically regulated their responses to unpleasant movies. Higher levels of VMPFC further impacted the regulation network associated with lower anger experience during the high-anger clips and lower trait anger levels (Jacob et al., 2018). These findings implicate frontal cortical mechanisms in emotion regulation, both provoked by non-naturalistic and naturalistic stimuli. Finally, a recent intracranial EEG study showed that the temporal pole regulates amygdala responses provoked by both pictures and movies (Sonkusare et al., 2020).

Distancing is another emotion regulation strategy that involves, for example, watching emotional pictures, with an understanding that they are from a movie rather than real. Ochsner and Gross (2008) consider distancing to be a subtype of reappraisal. Koenigsberg et al. (2010) found that distancing oneself from aversive images provoked an increased activation in dorsal ACC, medial prefrontal cortex, lateral prefrontal cortex, precuneus and posterior cingulate cortex, inferior parietal sulcus, and middle and superior temporal gyrus and decreased activation in the amygdala. Using EEG, Sulpizio et al. (2021) found a decrease in theta and beta bands in posterior regions. A multivariate pattern classification of fMRI data further revealed distributed patches of posterior cortical activation (Powers et al., 2020). Overall, distancing involves posterior brain areas in comparison to reappraisal or reinterpretation, which is more related to frontal areas.

## Concluding remarks

Recently, the neural basis of perspective taking has been studied with media-based stimuli such as movies and narratives. Looking across these studies, it seems that the brain flexibly recruits the areas that are needed given the requirements of a given perspective, however, inferior temporal-occipital areas and posterior-medial parietal areas seem to be engaged across perspectives. Studies conducted with precisely controlled stimuli and tasks help further clarify specific factors contribut ing to the recruitment of brain areas. For example, taking the visual perspective of another person seems to result in recruitment of TPJ. When putting oneself into the shoes of another person, similar to oneself, structures associated with autobiographical memory function are recruited, and when empathizing the pain of others is called upon, the affective parts of the pain matrix, that is AI and ACC, are recruited. While sensory aspects of perspective taking may lie in posterior regions, empathy and reappraisal are mainly provided by the lateral and medial prefrontal cortex. We envision that continued efforts of combining media-based and more traditional experimental designs, with the latter offering specific localizer tasks, will pave the way for a comprehensive understanding of the neural basis of perspective taking. Open questions include how the brain switches between perspectives across unfolding twists of a plot in a movie, and how fundamental attentional, memory, and executive processes relate to perspective taking, such as those utilized when actively inhibiting thinking of or remembering something (Anderson and Green, 2001; Nowicka et al., 2011; Detre et al., 2013; Marzi et al., 2014; Pierguidi et al., 2016). As a translational aspect, perspective taking may provide some emotion regulation strategies; for example, to decrease amygdala activity to negative stimuli as during emotional distancing (Koenigsberg et al., 2010).

## Author contributions

Both authors contributed equally to the writing of the mini-review manuscript. All authors contributed to the article and approved the submitted version.

## Funding

The work was financed by the Russian Science Foundation, project #22-48-08002, https://rscf.ru/project/22-48-08002/ and carried out using HSE Automated system of non-invasive brain stimulation with the possibility of synchronous registration of brain activity and registration of eye movements (Reg. num 354937).

## Conflict of Interest

The authors declare that the research was conducted in the absence of any commercial or financial relationships that could be construed as a potential conflict of interest.

## Publisher’s Note

All claims expressed in this article are solely those of the authors and do not necessarily represent those of their affiliated organizations, or those of the publisher, the editors and the reviewers. Any product that may be evaluated in this article, or claim that may be made by its manufacturer, is not guaranteed or endorsed by the publisher.
